# Validity of biopsy-based drug effects in a diet-induced obese mouse model of biopsy-confirmed NASH

**DOI:** 10.1186/s12876-019-1149-z

**Published:** 2019-12-28

**Authors:** Maria Nicoline Baandrup Kristiansen, Sanne Skovgård Veidal, Christina Christoffersen, Michael Feigh, Niels Vrang, Jonathan David Roth, Mary Erickson, Luciano Adorini, Jacob Jelsing

**Affiliations:** 1 Gubra Aps, Hoersholm, Denmark; 2 Department of Biomedical Sciences, Faculty of Health and Medical Sciences, University of Copenhagen, Copenhagen, Denmark; 3 Department of Clinical Biochemistry, Bispebjerg Hospital and Rigshospitalet, University of Copenhagen, Copenhagen, Denmark; 4 Intercept Pharmaceuticals, San Diego, California USA

**Keywords:** Nonalcoholic steatohepatitis, Stereology, Liver morphometry, Pharmacodynamics, Disease model, Liver biopsy

## Abstract

**Background:**

Compounds in clinical development for nonalcoholic steatohepatitis (NASH) improve liver histopathology in diet-induced obese mouse models of biopsy-confirmed NASH. Since the biopsy section used for histopathological evaluation represents only < 1% of the whole mouse liver, we evaluated how well biopsy-based quantitative image analyses correlate to stereology-based whole-liver quantitative changes upon drug treatment.

**Methods:**

Male leptin-deficient *Lep*^*ob*^*/Lep*^*ob*^ mice were fed the Amylin liver NASH (AMLN) diet for 16 weeks before stratification into treatment groups using a biopsy-based evaluation of type I collagen αI (col1a1) levels. Mice were treated for 8 weeks with either vehicle (PO, QD), liraglutide (0.4 mg/kg, SC, QD), elafibranor (30 mg/kg, PO, QD) or INT-767 (10 mg/kg, PO, QD). Terminal quantitative histological assessment of liver lipid (hematoxylin-eosin staining), inflammation (galectin-3 immunohistochemistry (IHC); gal-3), and fibrosis (col1a1 IHC) was performed on terminal liver biopsies and compared with stereologically sampled serial sections spanning the medial, left and right lateral lobe of the liver.

**Results:**

The distribution of liver lipid and fibrosis was markedly consistent across lobes, whereas inflammation showed some variability. While INT-767 and liraglutide significantly reduced total liver weight by 20 and 48%, respectively, elafibranor tended to exacerbate hepatomegaly in *Lep*^*ob*^*/Lep*^*ob*^-NASH mice. All three compounds markedly reduced biopsy-based relative liver lipid content. Elafibranor and INT-767 significantly reduced biopsy-based relative gal-3 levels (*P* < 0.001), whereas INT-767 and liraglutide tended to reduce relative col1a1 levels. When changes in liver weight was accounted for, both INT-767 and liraglutide significantly reduced biopsy-based total col1a1 content. Although minor differences in absolute and relative liver lipid, inflammation and fibrosis levels were observed across lobes, the interpretation of drug-induced effects were consistent with biopsy-based conclusions. Notably, the incorporation of changes in total liver mass revealed that liraglutide’s efficacy reached statistical significances for all analyzed parameters.

**Conclusions:**

In conclusion, in-depth analyses of liver homogeneity demonstrated that drug-induced improvement in liver biopsy-assessed histopathology is representative for overall liver effects assessed using stereology. Importantly, these findings reveal how changes in whole-liver mass should be considered to provide a deeper understanding of apparent drug treatment efficacy in preclinical NASH studies.

## Background

The prevalence of nonalcoholic fatty liver disease (NAFLD) is increasing worldwide alongside the increased incidences of diabetes and obesity [1, 2]. NAFLD ranges from benign nonalcoholic fatty liver (NAFL) with simple steatosis to the necroinflammatory state non-alcoholic steatohepatitis (NASH) and cirrhosis [3–5], which is estimated as the leading cause of end stage liver disease within a few years [6–8]. To date there is no licensed treatment for NASH, however, numerous clinical trials are ongoing [9]. Most advanced are obeticholic acid (a farnesoid X nuclear receptor (FXR) agonist), elafibranor (a dual peroxisome proliferator-activated receptor (PPAR)-α/δ agonist), selonsertib (an apoptosis signal-regulating kinase 1 (ASK1) inhibitor), and liraglutide (a long-acting glucagon-like peptide-1 (GLP-1) analogue) [9].

In addition to the difficult task of developing therapeutics for NASH, clinical diagnosis and follow-up data are hampered by the unmet need for reliable non-invasive diagnostic and prognostic tools [10, 11]. NASH development is unpredictable and vary in both disease severity and progression rates [12]. Non-invasive imaging procedures, including ultrasonography, magnetic resonance imaging (MRI) and magnetic resonance elastography (MRE) have shown potential in diagnosing NAFL and can be repetitively performed during the disease monitoring period [11]. However, their utility is inadequate due to a lack of sensitivity to differentiate between intermediate levels of fibrosis severity, their limited availability and associated costs [11]. Highly sensitive and predictive blood chemistry tests for circulating surrogate biomarkers of liver injury have still not reached FDA approval [11]. Accordingly, invasive and risky paired liver biopsies still remain the gold standard for staging and grading of NASH, and for monitoring drug efficacy in clinical trials [13–17].

To aid the development of pharmaceutical therapeutics, animal models reflecting the clinical NASH phenotype are of uppermost importance. Several models have been developed and are generally categorized into diet-induced, chemically-induced, or genetic models (knockout or transgenic) [18]. Different obesogenic Western-type diets have proven to promote a NASH phenotype in mice, though the disease severity is often mild [19]. However, when kept ≥26 weeks on a diet high in fat, fructose, and cholesterol (the Amylin liver NASH diet; AMLN [20, 21]), C57BL/6J mice have been shown to develop the hepatic pathological hallmarks of NASH, including steatosis, lobular inflammation, and ballooning degeneration, as well as mild to moderate fibrosis [21–26]. These hallmarks are further accentuated in leptin-deficient *Lep*^*ob*^*/Lep*^*ob*^ mice [20, 25, 27, 28]. The pharmacological efficacy on metabolic and hepatic endpoints have already been extensively characterized in these models [20, 26, 29].

Elafibranor, INT-767 and liraglutide have previously been shown to induce diverse pharmacodynamic effects on liver histopathology [20, 26, 29–34]. The three compounds represent three completely different drug classes with three different mechanism of action [29, 35–37] and are also known to affect total liver mass. While findings based on small tissue biopsies are encouraging, no studies have previously used gold standard stereological sampling to evaluate the homogeneity of liver morphometry across liver lobes nor to evaluate the validity of liver biopsy assessments to reflect pharmacologically induced changes on the whole mouse liver. This study aims to evaluate if biopsy-based quantitative image analysis efficiently reflects whole liver remodelling following drug treatment by comparison with stereology-based quantitative digital image analysis of the whole liver.

## Methods

### Animals and experimental set-up

Male B6.V-*Lep*^*ob*^/JRj (*Lep*^*ob*^*/Lep*^*ob*^) mice (5 weeks of age) were obtained from JanVier (JanVier Labs, France), and group housed 10 animals per cage in a controlled environment (12/12 h dark-light cycle, 21 ± 2 °C room temperature, and 50% ± 10% humidity). Mice had *ad libitum* access to the AMLN diet (D09100301, Research Diets, New Brunswick, United States) [21], containing 40% fat (18% trans-fat), 40% carbohydrates (20% fructose) and 2% cholesterol, or regular rodent chow (Altromin 1324, Brogaarden, Denmark), as well as tap water. Mice were kept on diet 16 weeks prior to an eight-week pharmaceutical intervention period (see below). Throughout the treatment period body weight was measured daily. All animal handling, treatments and euthanization were carried out according to the protocol approved by the Danish National Agency for Protection of Experimental Animals using internationally accepted principles for the care and use of laboratory animals (licence no. 2013-15-2934-00784, The Animal Experiments Inspectorate, Denmark).

### Pharmacological intervention

After 13 weeks on AMLN diet, a liver biopsy (pre-biopsy) was performed as described previously [21, 25, 26] for randomization and stratification. A priori histopathological inclusion criteria were a steatosis score ≥ 2 and a fibrosis stage score ≥ 1 as evaluated by one pathologist using the clinical criteria outlined by Kleiner et al [38]. Animals were single housed after the biopsy procedure. Following a three weeks recovery period, mice were stratified (*n* = 10–12 per group) based on mean quantification of type I collagen αI (col1a1). Mice were treated for eight weeks with INT-767 (Intercept Pharmaceuticals, San Diego CA, United States), liraglutide (Victoza™ pen) from Novo Nordisk (Bagsvaerd, Denmark), and elafibranor from SunshineChem (Shanghai, China). Vehicles were 0.5% carboxymethyl cellulose with 0.01% Tween-80 (per oral dosing; PO) or phosphate-buffered saline with 0.1% bovine serum albumin (subcutaneous dosing; SC), administered in a dosing volume of 5 mL/kg. Vehicle group (per oral dosing; PO, QD), INT-767 (30 mg/kg, PO, QD), liraglutide (0.2 mg/kg, SC, BID), or elafibranor (30 mg/kg, PO, QD). Vehicle-dosed chow-fed mice (PO, QD) served as additional controls. Animals were euthanized via exsanguination under general anaesthesia (induced by isoflurane (IsoFlo Vet, Orion Pharma, Denmark) O_2_ inhalation (2–4%)). This method was selected in order to simultaneously provide a large blood sample from each animal (1 ml of blood was drawn from the left ventricle using a vacutainer). Liver samples were processed as described below.

### Tissue processing and morphometric analyses

**Fig. 1:**
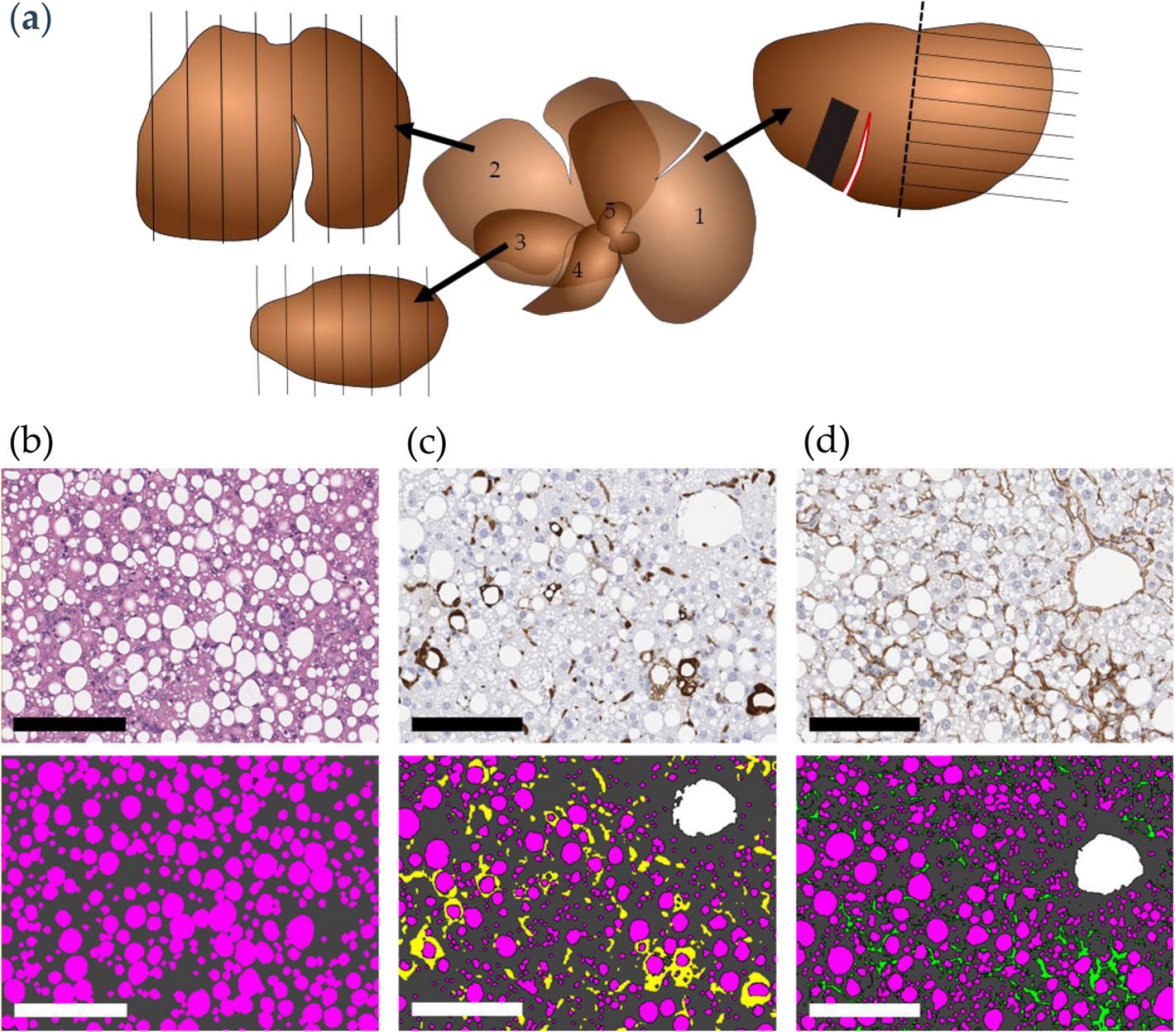
Visualization of stereological sections and morphometric analysis. Schematic drawing of liver lobes used for stereology (**a**); left lateral lobe (1), including site of pre-biopsy (red), post biopsy (black square), and stereology section (half the lobe marked by the dashed line), medial lobe (2), and right lateral lobe (3), as well as the caudate lobe (4) and papillary process (5). Morphometric analysis of; steatosis (**b**) on hematoxylin and Eosin Y stained sections, as well as immunohistochemistry (IHC) of galectin-3 (gal-3) (**c**), and of type I collage α1 chain (col1a1) (**d**). Pink is assigned to steatosis, dark grey assigned to liver tissue, yellow assigned to gal-3 positive stain, and green assigned to col1a1 positive stain. The morphometrical assessments are performed using Visiomorph Software (Visiopharm, Hoersholm, Denmark), by pixel annotation, excluding vessels based on size (white is assigned to vessels), as well as excluding gal-3 and col1a1 positive stain surrounding vessels. Scale bar = 150 μm

The whole liver was weighed and divided into the left lateral, medial, and right lateral lobe which were then weighed individually. Subsequently, the left lateral lobe was subdivided into two equal parts, from which a rectangular shaped terminal biopsy was obtained from one part. The remaining half, as well as the medial and right lateral lobes were used for stereology-based analyses (Fig. 1a-c).

All samples were immersion-fixed in 4% paraformaldehyde overnight followed by infiltration in paraffin. Liver pre- and terminal biopsies were embedded whole mount. The lobes used for stereology-based analyses were cut into thick (4 mm) systematic uniform random tissue sections (*n* = 6–8) using a razor blade fractionator as described previously [39], and embedded in blocks of paraffin cut-surface down. All blocks were sectioned into 3 μm paraffin sections on a microtome (Microme HM340E, Thermo Fisher Scientific, United Kingdom), and stained with Mayer’s Hematoxylin and Eosin Y (HE) (Dako, Glostrup, Denmark and Sigma-Aldrich, Broendby, Denmark), Picro-Sirius red (Sigma-Aldrich, Broendby, Denmark), anti-col1a1 (1:300; Southern Biotech, Birmingham; 2° antibody Bright Vision anti-goat, ImmunoLogic, Netherlands), or anti-galectin-3 (gal-3; 1:50000; Biolegend, San Diego, United States; 2° antibody anti-rat IgG 1:800, VWR, Soeborg, Denmark; Envision rabbit, Agilent Technologies, Glostrup, Denmark) according to standard procedures. Morphometric analyses of relative (area fraction) liver lipid, gal-3 and col1a1 levels were performed using Visiomorph software (Visiopharm, Hoersholm, Denmark) (Fig. 1). Estimates of total liver lipid, gal-3 and col1a1 were calculated using either biopsy-based assessments of relative values multiplied by the total liver weight or stereology-based assessments of relative values multiplied with the weight of the individual lobes.

### Statistical analysis

Statistical analysis was performed using either two-way analysis of variance (ANOVA) followed by Turkey’s post-hoc test, or one-way ANOVA with Dunnett’s post-hoc test (*p* < 0.05 was considered statistically significant). The variance of measurement of the stereological sections form the individual lobes were analysed using the coefficient of variation (CV). All data are presented as mean ± standard error of the mean (SEM).

## Results

### Intra- and interlobular variability

**Fig. 2:**
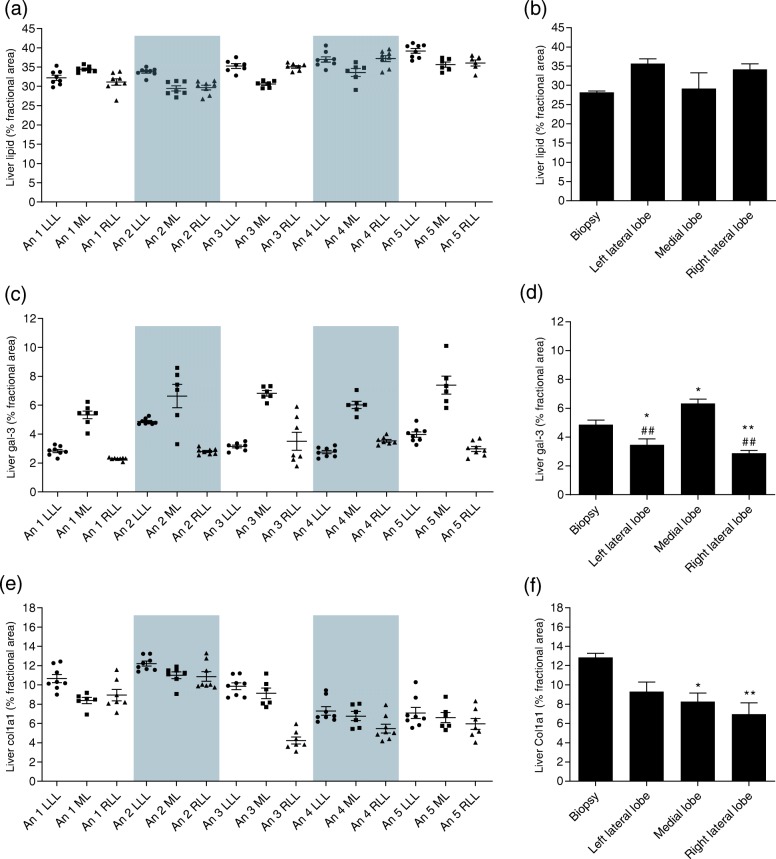
Assessment of intra- and interlobular variability Quantification of liver lipids, galectin-3 (gal-3), and type I collagen α1 chain (col1a1) as determined by morphometry of stereological sections and terminal biopsy. Varity of sections used for stereological assessment of liver lipids (**a**), gal-3 (**c**), and col1a1 (**e**) assessed in left lateral lobe (LLL), medial lobe (ML), and right lateral lobe (RLL) from five NASH Vehicle animals. An 1 = animal 1, An 2 = animal 2 and so forth. Quantification of terminal biopsy, mean of sections from LLL (**b**), ML (**d**), and RLL (**f**) from five NASH vehicle animals. Data expressed as mean ± SEM (*n* = 6–8). **P < 0.01 vs. Biopsy, *P < 0.05 vs. Biopsy, ^##^*P* < 0.01 vs. Medial lobe, ^#^*P* < 0.05 vs. Medial lobe. One-way ANOVA with Turkey’s multiple comparison test

The intra- and interlobular variability was analyzed in five NASH vehicle-treated mice, as power calculations estimated this sufficient. The morphometric analyses of liver lipid revealed a low intra-lobe variability (CVi of 0.02 to 0.08) and a slightly higher variability across lobes (inter-lobe CV of 0.06–0.08) (Fig. 2a). Biological variability between animals accounted for 43.7% of the total variance, whereas intra-lobe variability accounted for only 11.7%. No significant differences in liver lipid was observed between biopsies and individual lobes (Fig. 2b).

The most conspicuous differences in intra-lobe variability was seen for gal-3 morphometry with a CVi of 0.04 to 0.47 (Fig. 2c). Moreover, gal-3 levels were significantly higher in the medial lobe, as compared to the left and right lateral lobe, and as compared to the biopsy-based gal-3 levels (Fig. 2d). Biological variability accounted for 8% of the total gal-3 variance, whereas intra-lobe variability accounted for 67.8%.

Intra-lobe variability for col1a1 was estimated to 0.06–0.26 (Fig. 2e). As for liver lipid content, the largest discriminator for variance of col1a1 was biological variability among mice, accounting for 52.9% of the total variance, whereas intra-lobe variance accounted for only 14.7%. In general, estimates of total col1a1 levels in individual lobes were significantly lower than biopsy-based assessments (Fig. 2f).

### Effects on body and liver weight

**Fig. 3:**
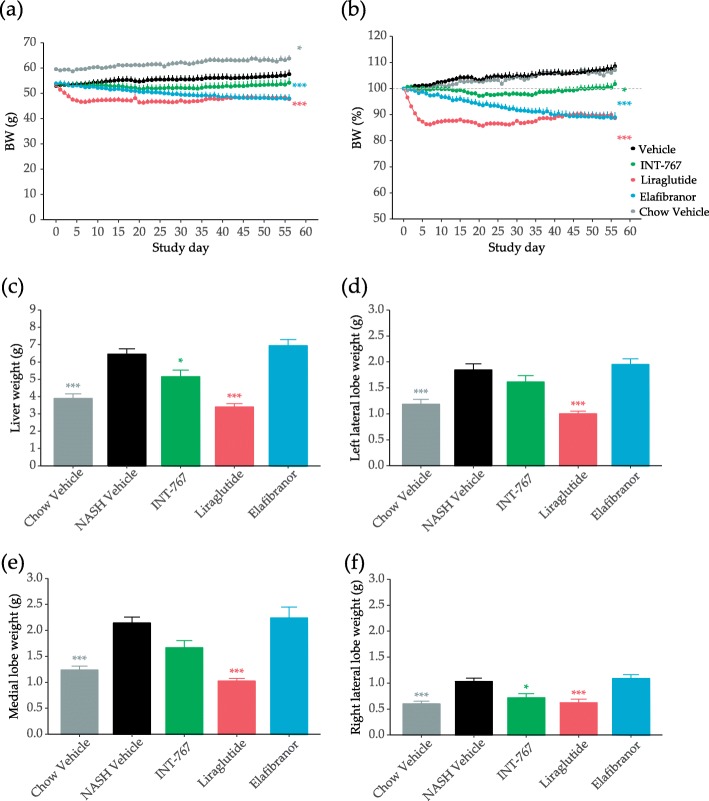
Treatment effect on bodyweight and liver weight. Bodyweight (**a**) and bodyweight change (**b**) during the study period. Liver weight (**c**), weight of lobes used for stereology; left lateral lobe (**d**), medial lobe (**e**), and right lateral lobe (**f**). Data expressed as mean ± SEM (*n* = 10–12). ***P < 0.001 vs. Vehicle. One-way ANOVA with Dunnett’s multiple comparison test

*Lep*^*ob*^*/Lep*^*ob*^-NASH groups were obese prior to treatment (53 ± 1.1 g, *n* = 12), but showed lower body weight compared to age-matched chow-fed *Lep*^*ob*^*/Lep*^*ob*^ vehicle-treated mice (59.6 ± 0.8 g, *n* = 10) (Fig. 3a). Liraglutide and elafibranor treatment progressively reduced body weight (Fig. 3a), with a maximal weight loss of approximately 10% compared to baseline, and approximately 20% vs vehicle-dosing (Fig. 3b). INT-767 slowed the rate of body weight gain, but did not reduce body weight below baseline levels in *Lep*^*ob*^*/Lep*^*ob*^-NASH mice (Fig. 3b).

*Lep*^*ob*^*/Lep*^*ob*^-NASH mice had marked hepatomegaly compared to chow fed controls (Fig. 3c-f). Both liraglutide and INT-767 significantly reduced total liver weight (Fig. 3c) and right lateral lobe weight (Fig. 3f). A similar pattern was observed for the left lateral and medial lobes achieving statistical significance for liraglutide only (Fig. 3d and e). Elafibranor treatment did not significantly affect liver weight.

### Analyses of terminal biopsy and whole liver morphometry

**Fig. 4:**
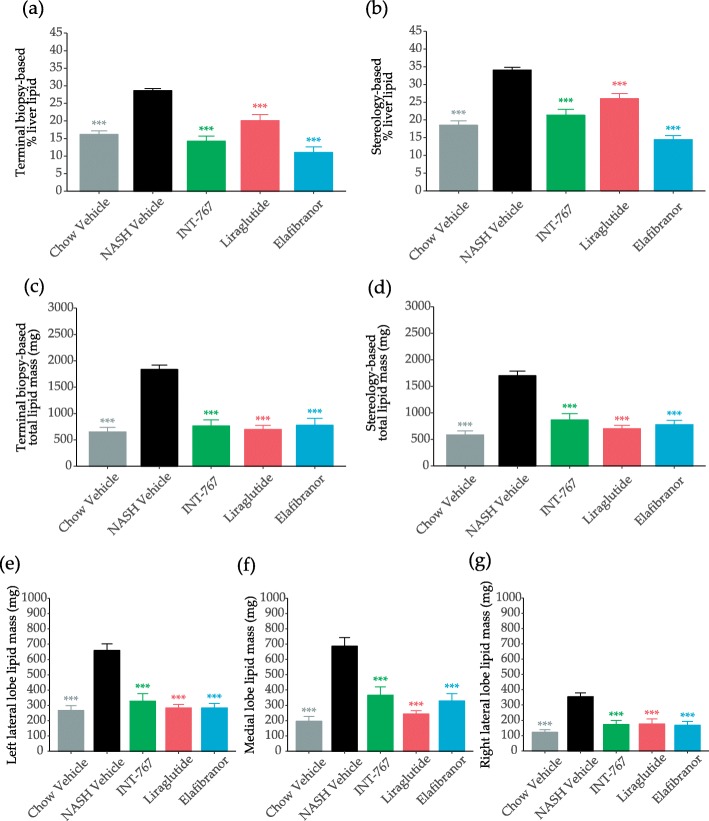
Morphometric quantification of liver lipids. Terminal relative liver lipid quantification as determined by morphometry of liver biopsy (**a**), terminal relative liver lipid quantification as determined by stereology (**b**), terminal total lipid mass as determined by morphometry of liver biopsy (**c**), and terminal total lipid mass as determined by stereology (**d**),as well as terminal total lipid mass as determined by stereology of left lateral (**e**), medial (**f**), and right lateral lobe (**g**). Data expressed as mean ± SEM (n = 10–12). ***P < 0.001 vs. Vehicle. One-way ANOVA with Dunnett’s multiple comparison test

All treatments significantly reduced relative lipid content in the biopsy (Fig. 4a) and in the whole liver (Fig. 4b), being most pronounced for INT-767 and elafibranor. When incorporating changes in overall liver weight, the effect on total lipid mass was even more conspicuous irrespective of biopsy (Fig. 4c) or whole liver (Fig. 4d) based sampling. This was also evident from the sub-analyses of individual lobes (Fig. 4e-g, Table 1).

**Table 1:** Analysis of % decrease of liver lipid, galectin-3 (gal-3), and type I collage α1 chain (col1a1). Decrease was analyzed based on levels of the respective NASH vehicle group for terminal biopsy, whole liver, left lateral, medial and right lateral lobe. Data expressed as mean percentage (*n* = 10–12)

Analysis mass (mg)	Group	Biopsy % decrease	Whole liver % decrease	Left lateral lobe % decrease	Medial lobe % decrease	Right lateral lobe % decrease
Liver lipid	INT-767	58	49	50	47	55
	Liraglutide	62	59	57	65	51
	Elafibranor	58	54	57	52	53
Gal-3	INT-767	54	58	51	62	58
	Liraglutide	47	46	41	52	35
	Elafibranor	38	46	48	45	45
Col1a1	INT-767	38	58	61	55	57
	Liraglutide	41	38	42	38	24
	Elafibranor	11	26	24	29	25

**Fig. 5:**
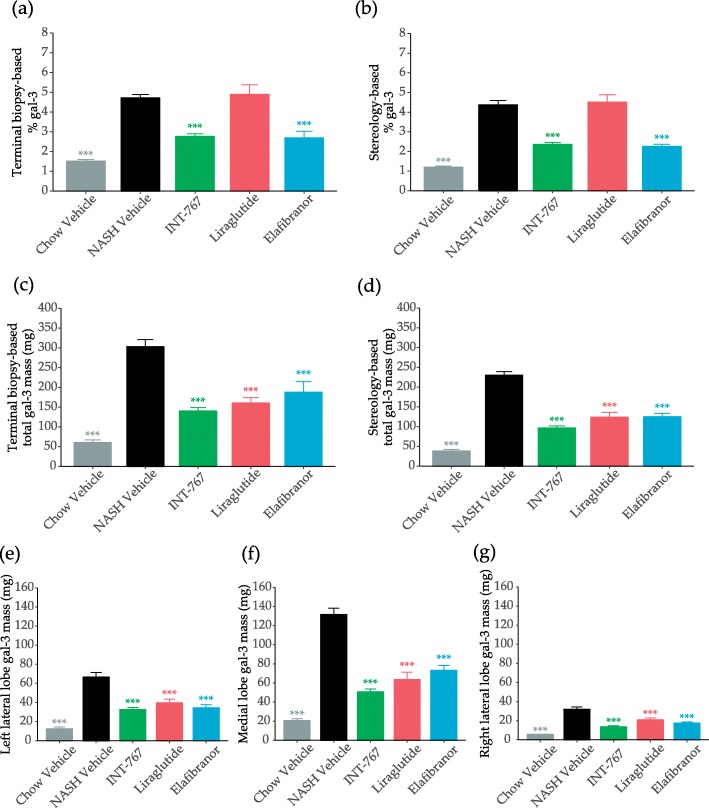
Morphometric quantification of galectin-3 (gal3). Terminal relative gal-3 quantification as determined by morphometry of liver biopsy (**a**), terminal relative gal-3 quantification as determined by stereology (**b**), terminal total gal-3 mass as determined by morphometry of liver biopsy (**c**), terminal total gal-3 mass as determined by stereology (**d**), as well as terminal total gal-3 mass as determined by stereology of left lateral (**e**), medial (**f**), and right lateral lobe (**g**). Data expressed as mean ± SEM (*n* = 10–12). ***P < 0.001 vs. Vehicle. One-way ANOVA with Dunnett’s multiple comparison test

INT-767 and elafibranor significantly reduced relative gal-3 levels in the biopsy (Fig. 5a) and in the whole liver (Fig. 5b), whereas liraglutide had no effect on relative gal-3 levels (Fig. 5a-b). However, when expressed as total values, all compounds reduced gal-3 mass irrespective of sampling method (Fig. 5c-d), and across all lobes (Figs. 5e-g, Table 1).

**Fig. 6:**
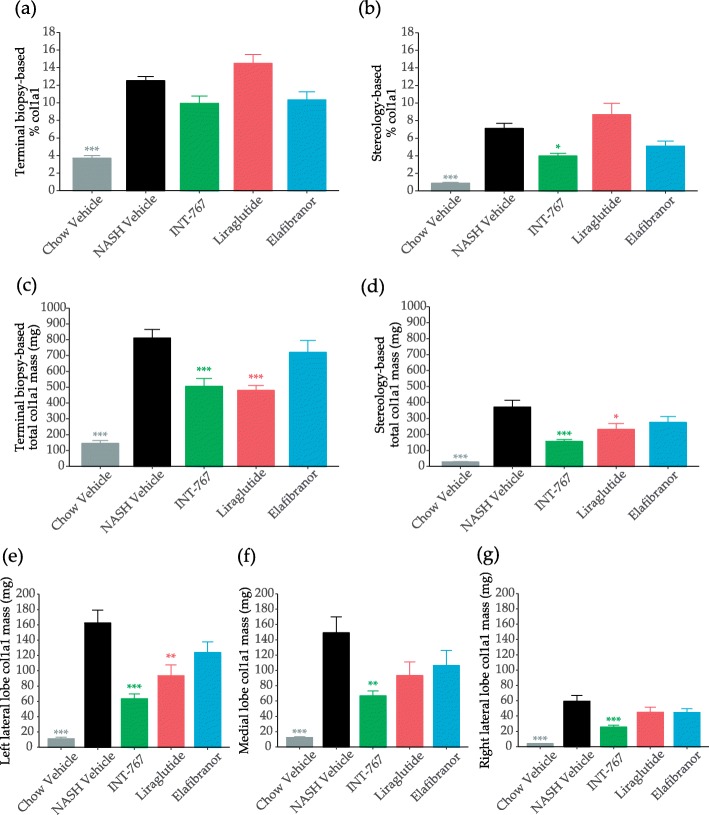
Morphometric quantification of type I collage α1 chain (col1a1). Terminal relative col1a1 quantification as determined by morphometry of liver biopsy (**a**), terminal relative col1a1 quantification as determined by stereology (**b**), terminal total col1a1 mass as determined by morphometry of liver biopsy (**c**), and terminal total col1a1 mass as determined by stereology (**d**), as well as terminal total col1a1 mass as determined by stereology of left lateral (**e**), medial (**f**), and right lateral lobe (**g**). Data expressed as mean ± SEM (*n* = 10–12). *P < 0.05, **P < 0.01, ***P < 0.001 vs. Vehicle. One-way ANOVA with Dunnett’s multiple comparison test

None of the treatments effectively reduced biopsy-based relative col1a1 (Fig. 6a), whereas INT-767 reduced whole liver relative col1a1 (Fig. 6b). When expressed as total values, INT-767 and liraglutide, but not elafibranor, significantly reduced total liver col1a1 levels irrespective of sampling method, i.e. biopsy- (Fig. 6c) or stereology-based quantitation (Fig. 6d). Only INT-767 significantly reduced col1a1 mass across all lobes (Figs. 6e-f) and exerted the most pronounced col1a1 reducing effect among the drug classes tested (Table 1). In general, the reduction in col1a1 mass was higher in stereology-based analysis compared to biopsy-based analysis.

## Discussion

The present study aimed to verify the validity of a liver biopsy, representing less than 1 % of the total liver, to reflect whole liver disease remodeling following pharmaceutical treatment in male *Lep*^*ob*^*/Lep*^*ob*^-NASH mice. By comparing morphometric analyses on biopsies with stereologically sampled sections across the whole liver, we demonstrate that the biopsy is overall representative of the whole liver status and is applicable for preclinical evaluation of pharmacological intervention studies. Notably, however, we also demonstrate that pharmacologically induced effects on liver weight should be carefully considered when comparing NASH related endpoints in preclinical studies.

Whereas liver lipid content showed little variation within and between lobes, intra-lobe variability was more evident for both relative gal-3 and col1a1 levels. This difference is not surprising and emphasizes the need to take biopsies in the same part of the lobe when comparing tissue dynamics between different animals, or to use unbiased stereological sampling principles covering the whole liver. The differences in col1a1 levels is mainly related to the fraction of Glisson’s capsule in the tissue section. The Glisson’s capsule [40], a collagenous layer covering the liver, increases in thickness during progression of fibrosis [41, 42]. Accordingly, levels of col1a1 was markedly higher at apical parts compared to slaps containing a higher ratio of central parts of the lobe. The same reason may apply for the variability of gal-3, as, macrophage-derived gal-3 is known to be linked to myofibroblasts and hence fibrosis [43, 44].

In addition to the in-depth assessment of lobe variability and the validation of liver biopsy assessments in mice, we characterized the effects of liraglutide, elafibranor, and INT-767 in *Lep*^*ob*^*/Lep*^*ob*^-NASH mice. Liraglutide, a human GLP-1 analogue, is already FDA approved for the treatment of obesity (Saxenda®) and type 2 diabetes (Victoza®) [45, 46], and is in addition to it's well-described incretin effects [47], also reported to improve liver enzymes, oxidative stress, and steatosis [20, 26, 30, 31, 37, 48]. In contrast, elafibranor, a high-affinity agonist for PPAR-α/δ, exerts its effect on NASH amelioration mainly by increasing clearance of fatty acids, as well as inhibition of pathways involved in inflammation and fibrosis [26, 33, 34]. Finally, INT-767, a dual FXR and transmembrane G-protein-coupled receptor 5 (TGR5) agonist, dose dependently reduce cholesterol and liver triglyceride levels, reduce steatosis, inflammation, and fibrosis stage [29, 32]. In human liver and plasma samples both FXR and TGR5 levels correlates with NAFLD disease severity [49–51]. All three compounds exerted marked effects on relative liver lipid content, whereas only INT-767 and elafibranor affected relative gal-3 levels. Only stereology-based assessment of INT-767 efficacy revealed improvements on relative col1a1 levels. However, when incorporating compound specific effects on liver size, both INT-767 and liraglutide significantly improved liver fibrosis, as well as total liver lipid and inflammation. In contrast, elafibranor did not reduce total col1a1, as also reported previously in both C57bl/6 and *Lep*^*ob*^*/Lep*^*ob*^ mice [26]. Thus, the presented data highlight the importance of looking at whole organ dynamics, instead of reporting relative values. Since liraglutide and INT-767 significantly reduce liver weight, mainly by reducing lipid content, relative values of col1a1 and gal-3 content would tend to show no regulation or even upregulation if not affected directly by the compound. Conversely, the peroxisome proliferating mechanism of elafibranor, which may lead to hepatomegaly in rodent models of NASH [26], would indirectly lead to biased reduced relative values of all other liver components if not addressed directly.

It should be noted that the comparison was based on image analyses and not a histopathological assessment of NAFLD activity scores and fibrosis stage, as reported previously [26]. Image analysis allows for an objective analysis of the liver histomorphology, whereas scoring and staging by a trained pathologist is more subjective. Image analysis of relative hepatic lipid levels is based on the actual amount of lipids in a histologic section (i.e. area or volume fractions) [52], whereas steatosis scores are graded based on the percentage of hepatocytes having lipid droplets, irrespective of the size of the lipid droplets [38]. Similarly, staging of fibrosis is based on the localizations of fibrotic bands, and not the area or thickness of fibrotic bands which is estimated in image analysis [25, 26, 53]. Lastly, scoring of lobular inflammation depends on the number of inflammatory foci (clusters of inflammatory cells) in 200X field of view [38], and not the total content of inflammatory cells (here assessed by gal-3 IHC). Thus, image analyses of NASH components are not necessarily directly correlated to histopathological scoring and staging. This inherent variability is an appropriate feature of clinical studies where only a small fraction of the organ can be sampled. However, at the preclinical stage, when one is trying to differentiate compounds within or across modalities it is readily possible to gain a more accurate assessment of the true disease state of the total organ.

Finally, it should be stated that the analyses presented here were based on a quantitative assessment of gal-3 and col1a1 immunohistochemistry. These “pan-markers” of inflammation and fibrosis are used extensively in preclinical and clinical research but may of course not represent all inflammatory of extracellular matrix remodelling during NASH development. Accordingly, the validity of biopsy-based drug efficacy presented here should be considered cautiously for other markers.

## Conclusion

In conclusion, we report that a liver biopsy can be considered representative for the remodeling occurring in the entire liver of *Lep*^*ob*^*/Lep*^*ob*^-NASH mice following pharmaceutical treatment, though changes are slightly different using an in-depth stereological assessment of the whole organ, as evidenced here for liraglutide and INT-767 for col1a1 assessments. In a recent study, repeated liver biopsies were extracted from the left lateral lobe, medial right lobe and medial left lobe in mice over a period of three months [54]. Although these data showed that repeated liver biopsies from different lobes were feasible, our data suggests that continuous biopsy-based measurements should be performed in the same lobe. Finally, we highlight the importance of introducing effects on total liver remodeling when assessing liver histomorphometry, as clearance of steatosis or hepatomegaly would bias relative values.

## Abbreviations

AMLN: Amylin liver nonalcoholic steatohepatitis
ANOVA: Analysis of variance
ASK1: Apoptosis signal-regulating kinase 1
Col1a1: Type I collagen αI
CV: Coefficient of variation
FXR: Farnesoid X nuclear receptor
Gal-3: Galectin-3
GLP-1: Glucagon-like peptide-1
HE: Hematoxylin and Eosin Y
IHC: Immunohistochemistry
*Lep*^*ob*^*/Lep*^*ob*^: B6.V-*Lep*^*ob*^/JRj leptin-deficient mice
MRE: Magnetic resonance elastography
MRI: Magnetic resonance imaging
NAFL: Nonalcoholic fatty liver
NAFLD: Nonalcoholic fatty liver disease
NASH: Nonalcoholic steatohepatitis
PO: Per oral dosing
PPAR-α/δ: Peroxisome proliferator-activated receptor-α/δ
SC: Subcutaneous dosing
SEM: Standard error of the mean
TGR5: Transmembrane G-protein-coupled receptor 5
